# Exploring the equity of distribution of general medical services funding allocations in Wales: a time-series analysis

**DOI:** 10.3399/BJGPO.2024.0080

**Published:** 2025-03-26

**Authors:** Jonny Currie, Kathrin Thomas, Anne M Cunningham, Kerry Bailey, Haroon Ahmed, Daniel Farewell, Sally Lewis

**Affiliations:** 1 Division of Population Medicine, Cardiff University, Cardiff, UK; 2 Department of Health, Belfast, Northern Ireland; 3 Primary Care Division, Public Health Wales, Cardiff, UK; 4 Value in Health, NHS Wales, UK

**Keywords:** inequalities, health inequities, statistics, Wales, primary health care

## Abstract

**Background:**

Good access to quality primary care in high-income countries can improve population health. Access to primary care is, however, often not equal among socioeconomic groups; our analysis sought to explore whether funding, a determinant of service supply, is equitably distributed among GP practices in Wales.

**Aim:**

To explore the relationship between funding and deprivation among GP practices in Wales, to understand the equity of current funding policies.

**Design & setting:**

A time-series analysis was undertaken in the primary care setting in Wales.

**Method:**

We obtained funding data for general practices in Wales between 2014 and 2022, and explored the equity of distribution using the percentage of practice patients living in the 20% most deprived small areas in Wales. We generated a linear regression model exploring the relationship between practice funding and deprivation, with an interaction term with time in years.

**Results:**

Practice funding rose for all practices between 2014 and 2022. Practice deprivation and time in years were both associated with practice funding, with increases in practice deprivation associated with reduced funding allocations, and time being associated with a small increase in funding over the study period. Over the period of analysis of 2014–2022, for every 10% increase in patients living in the most deprived lower layer super output areas, funding per patient decreased on average by 1%.

**Conclusion:**

General practices in Wales in more deprived areas receive discernibly less funding per patient than those in less deprived areas. Given the potential and likelihood primary care can affect population health outcomes, this underinvestment may be contributing to existing health inequalities and requires urgent further analysis and action.

## How this fits in

Primary care has the potential to mitigate health inequalities through comprehensive, accessible, and quality care. Previous analyses of GP funding in the UK have found little to no variation at a population level between people living in the most and least deprived areas. This analysis of funding at a practice level shows clear evidence of inequitable funding: for every 10% increase in patients at a GP practice from the most deprived areas in Wales, practices received 1% less funding. Clinicians practising in areas of high deprivation could benefit in recruitment, retention, and service delivery were funding to be more equitably distributed.

## Introduction

Good access to quality primary care in high-income countries can improve population health.^1,2^ Primary care services that are well-resourced and provide continuity of care between primary care professionals and patients have been shown to reduce mortality,^3,4^ hospital admissions,^5–7^ and costs.^8^

Access to primary care has been shown to have differential impacts between socioeconomic groups. One study found access to primary care attenuated the relationship between income inequality and self-reported health,^9^ while availability of GPs may, in more deprived areas, be associated with lower emergency hospital admissions.^10^ Access to primary care is, however, often not equal among socioeconomic groups; in England, supply of GPs, numbers of patients registered per practice, and uptake of appointments have all been shown to favour less deprived areas,^11,12^ which is evidence of the so-called ‘inverse care law’, first coined by Julian Tudor Hart in 1971, that the availability of good medical care tends to vary inversely with need.^13^ Healthcare needs are greater in more deprived communities because people become more unwell with multimorbidities at younger ages and would be expected to have greater healthcare utilisation if supply was matched to need.^14^

Among factors that determine the supply and quality of primary care services, funding is a key determinant. Funding for primary care in UK nations is from government through general taxation, similar to some other high-income countries.^15^ Funding in the UK is allocated using an allocation formula.^16^ Although primary care funding formulae across the UK account for deprivation, research published in Scotland^17^ and England^18^ suggests existing formulae do not sufficiently account for levels of unmet need in areas of greater deprivation.

Enduring levels of income inequality, the legacy of deindustrialisation, and other factors in Wales have led to persisting inequalities in life expectancy and health. Wales has two main patterns of deprivation: urban deprivation in the cities in the south and north-east of the country, and the post-industrial communities of the Welsh Valleys, with areas of deprivation largely concentrated in four of the seven health board areas. Women and men in the most deprived parts of Wales live an average of 6 years and 7 years less than those in the least deprived parts, respectively,^19^ with such gaps continuing to grow over time. Little research is published on the levels of available funding for primary care in Wales by level of area deprivation. To our knowledge, no studies have assessed the drivers of maldistribution of primary care services in Wales, particularly funding as determined by national allocation formulae. In this study, we report an evaluation of funding to primary care in Wales by area deprivation at a practice level, to explore persisting inequalities presence of the inverse care law.

## Method

### Data sources

**Figure 1.**
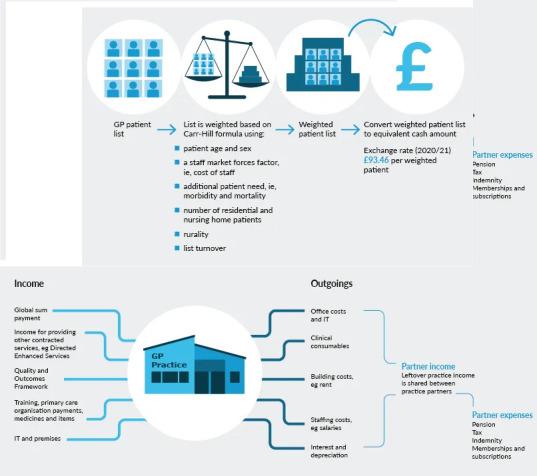
Infographics summarising factors used to inform GP practice funding in the Carr–Hill formula. The lower infographic illustrates the wider revenue streams received by GP practices in Wales, with Quality and Outcomes Framework funding now representing Quality Assurance and Improvement Framework monies. Reused with permission from The King's Fund.^29^

We obtained funding data for general practices in Wales between 2014 and 2022 from the NHS Wales Shared Services Partnership. General practices in Wales receive funding via a number of revenue streams: a capitated budget is calculated based on the patient demographic of a practice and other geographic factors as per the Carr–Hill formula,^20^ to which additional funding is generated ranging from enhanced services, premise costs, performance-related payments, and other costs (see Figure 1). We annualised funding data from quarterly datasets and calculated allocations per patient using the median list size for each year period, given that population sizes significantly affect practice funding. We used a lookup of general practice deprivation indices published in 2022 involving the percentage of registered patients living in the 20% most deprived local areas.^21^

### Data analysis

Funding data were available by year, practice, and revenue stream. We linked funding and practice deprivation data using practice identifier codes. Funding data were adjusted for inflation during the period using indices from the UK HM Treasury department.^22^ We explored trends in funding data and the distribution of our outcome and explanatory variables before statistical analysis; both funding and practice deprivation data were significantly right skewed, leading us to transform funding per patient per year at practice level through the natural logarithm; we left practice deprivation as an explanatory variable untransformed. We generated a linear regression model, as illustrated below, with an interaction term between time in years since 2014 and practice deprivation, allowing for a change over time of the relationship between funding and deprivation. Analysis was undertaken in RStudio (version 2023.09.1) using the *tidyverse*, *knitr*, and *broom* packages. The below equation, a linear regression model equation, was used, exploring the association between GP practice funding in Wales and practice deprivation, expressed in Wilkinson–Rogers:^23^


$$\begin{array}{l} \log\left(\text{total practice funding}\right)\\ \;\;\;\;=\left(\text{year since 2014}\right)*\left(percentage\;of\;patients\;in\;20\%\;most\;deprived\;\text{LSOAs}\right) \end{array}$$

## Results

**Figure 2.**
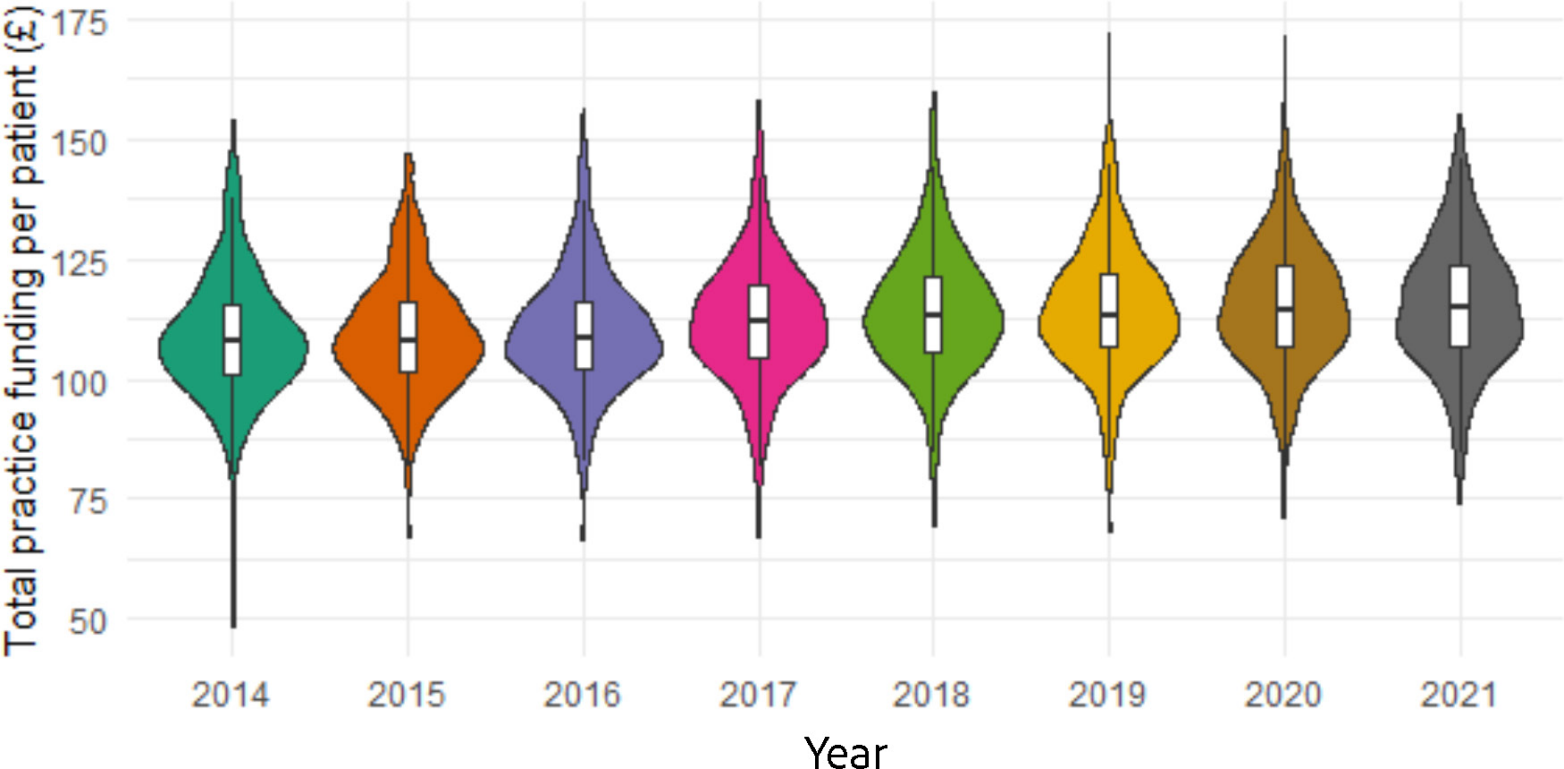
Chart showing median and interquartile ranges (small boxplots) of GP practice funding for years 2014–2015 to 2021–2022, with distribution of funding (violin plots) in each year period across all practices. Source: NHS Wales Shared Services Partnership, 2023, unpublished data.

Funding data were available for 384 practices for the study period of 2014–2022, distributed fairly evenly across the socioeconomic gradient and out of a total of 390 practices available from the 2022 practice deprivation lookup. Figure 2 presents a summary of trends in general practice funding in Wales for the study period. Median practice funding in 2014–2015 was £108 per patient (interquartile range [IQR] £93–£122), reaching the maximum in 2021–2022 at £115 per patient (IQR £98–£131).

**Figure 3.**
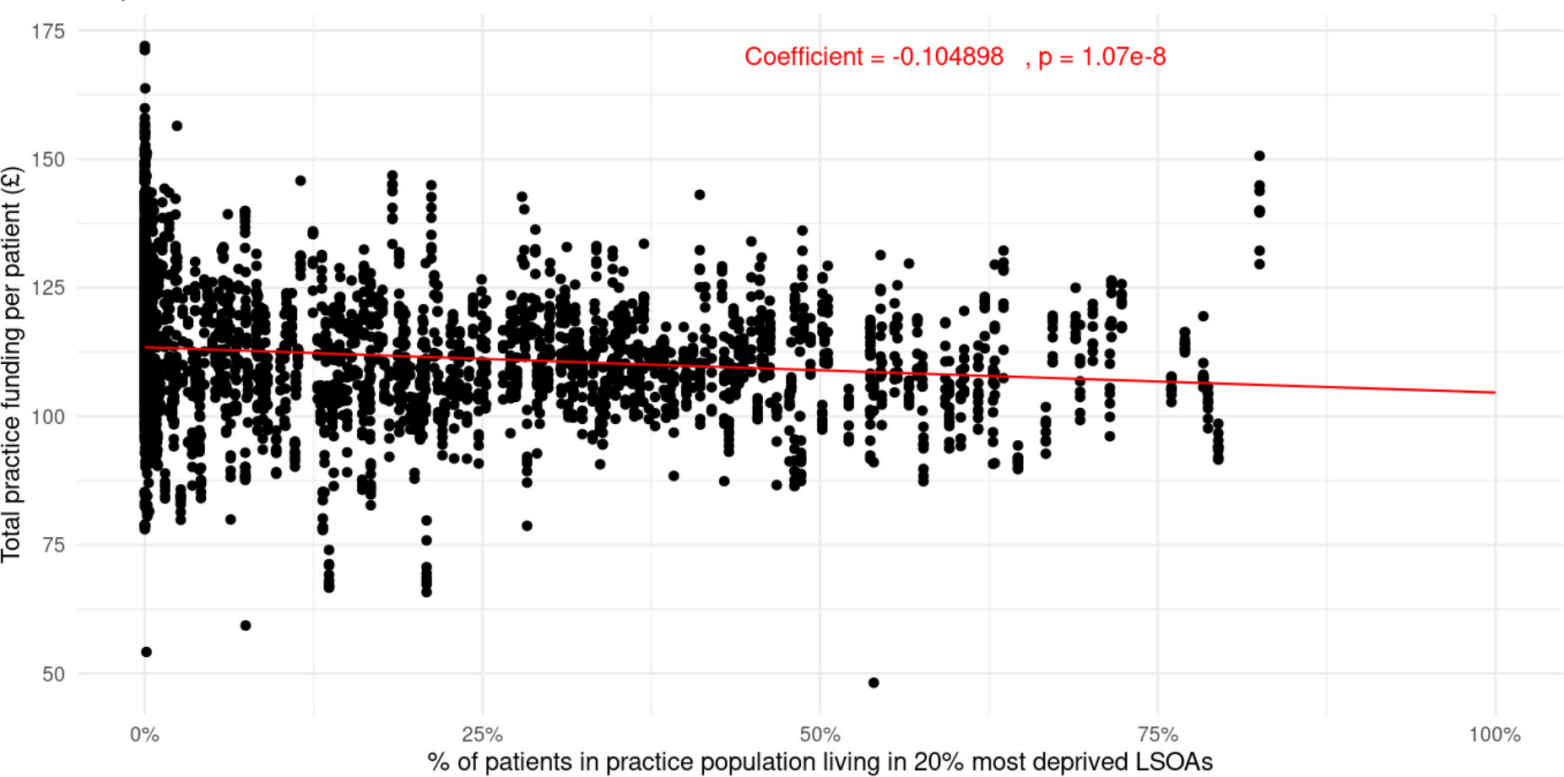
Scatterplot showing relationship between practice population deprivation and total practice funding in GP practices in Wales, 2014–2022. LSOA = lower layer super output area.

Table 1 summarises the linear regression model output exploring the association between GP practice funding in Wales and practice population deprivation indices. Practice deprivation and time in years were both associated with practice funding, with increases in practice deprivation associated with reduced funding allocations, and time being associated with a small increase in funding over the study period. Both associations were statistically significant, with *P*-values well below the 5% level. Over the period of analysis of 2014–2022, for every 10% increase in patients living in the most deprived lower layer super output areas (LSOAs), funding per patient decreased on average by 1%. Figure 3 illustrates the model output in graphical form, highlighting the relationship specifically between practice deprivation and funding.

**Table 1.** Regression model output: relationship between GP practice funding, practice deprivation, and time in GP practices in Wales, 2014–2022

Term	Coefficient	Standard error	*P* value
Intercept	4.700	0.005	<0.01
Practice deprivation	-0.105	0.018	<0.01
Year	0.009	0.001	<0.01
Practice deprivation: year interaction	0.007	0.004	0.114

## Discussion

### Summary

This study sought to explore relationships between GP practice funding in Wales and deprivation. Over the study period of 2014–2022, we found a small but significant association between the proportion of patients registered at a GP practice living in the 20% most deprived LSOAs and lower overall funding allocations to these practices. Such an association has persisted despite some increases in overall funding to general practices over the study period. Further analysis is required to contextualise such findings and consider wider factors affecting primary care workload and performance, including workforce issues, demand levels, prevalence of long-term conditions, and practice population age structures. However, our findings appear to confirm the presence of a statistically discernible underinvestment in primary care in areas of greater deprivation.

### Strengths and limitations

Our study findings are strengthened by several methodological factors. First, we collated funding data on a large number of practices, spanning the full range of General Medical Services revenue. Second, we conducted our analysis at the practice level, avoiding any statistical dilution of effect, and adjusted for practice population size. Finally, we adjusted for inflation, recognising funding allocations in one year cannot be compared across a longer period without such adjustments.

Our study has some limitations. Practice deprivation scores were based on registered patients: it has been recognised for some time that datasets of registered patients suffer from inaccuracies,^24^ which could render such deprivation data less robust, although it is unclear in which direction such biases could run. Furthermore, were certain practices (for example, those serving more deprived areas) at greater risk of inaccuracies, this could bias the wider analysis. Our analysis does not explore further factors relevant to practice performance alongside funding; further research is necessary to understand the entire landscape of factors affecting the equitable delivery of primary care services in Wales. We were only able to analyse practices based on their deprivation scores in 2021; practices in Wales in 2014 were of far greater number potentially biasing our results by failing to account for practices that closed or merged during the study period.

### Comparison with existing literature

Our study confirms wider literature from across the UK highlighting inequities arising from general practice funding formulae and the Carr–Hill method. A study by McLean and colleagues^17^ in Scotland examined funding in 2011–2012 and found a flat gradient, with little evidence that funding was adjusted sufficiently for deprivation. No analysis was undertaken to identify any statistical relationship between funding and practice deprivation. The study was cross-sectional covering 2011–2012 only and the authors aggregated practices into deprivation deciles, which may have masked any gap in funding between practices.

Fisher and colleagues^11^ undertook a similar analysis in 2020 of English general practice funding, aggregating practices to five areas of deprivation quintiles. The authors report, similar to the Scottish study, a flat gradient in funding between practice quintiles, suggesting little significant adjustment for deprivation. This study again used cross-sectional data from 2018–2019, aggregated funding data without allowing for an association at a practice level, and did not explore any statistical relationship between funding and practice deprivation. As such, we believe our study is the first to conclude with confidence that there appears to be a clear inequity at practice level in Wales, driven by the current Carr–Hill funding formula, which has been in place since 2004.

### Implications for research and practice

We believe the finding that GP practices in Wales in more deprived areas receive less funding to be inherently inequitable. We can speculate that such funding inequalities may have negatively affected service delivery in more deprived areas in Wales, and risk poor access or even quality of care for patients in more deprived areas. This is further affected by the challenges in recruiting staff to areas of higher deprivation.^25^ Further research is needed to understand the wider equity of primary care service delivery in Wales, reviewing how current funding is informing workforce recruitment across the gradient, the levels of workload on services, and what, if any, different outcomes are being realised in primary care quality and population health. Analysis considering other factors such as urban and rural geographies or the impact of dispensing status, where practices can earn further income from the provision of prescription medicines, may provide further insights. Further research, however, is complicated in Wales by a lack of available data, particularly since the cessation of reporting through the Quality and Outcomes Framework. In England, data are available on primary care appointment demand,^26^ long-term condition prevalence and management,^27^ and patient satisfaction,^28^ with data available either at a practice or regional level, allowing some exploration of variation by area deprivation. Data are sparse on the above domains in Wales, enabling little in the way of evaluation of the effectiveness nor equity of the NHS primary care system. While recognising wider debate on the levels of funding primary care requires to deliver quality services is ongoing, we believe policymakers and the profession must engage on the equity of current financing arrangements, ensuring that those patients who stand either most to gain or to lose (given wider social inequalities in our society) are prioritised.

In conclusion, general practices in Wales in more deprived areas receive discernibly less funding per patient than those in less deprived areas. Unless the inverse care law is to endure for further generations, Wales is in dire need of a new funding formula that recognises the need to better account for deprivation. Given the likelihood that primary care can affect population health outcomes, this underinvestment may be contributing to existing health inequalities and requires urgent further analysis for the NHS in Wales and policymakers to understand any patient safety and public health impacts.

## Data

The dataset relied on in this article is available from the corresponding author on reasonable request.

## References

[1] Starfield B, Shi L, Macinko J (2005). Contribution of primary care to health systems and health. Milbank Q.

[2] Gulliford M (2017). Access to primary care and public health. Lancet Public Health.

[3] Pereira Gray DJ, Sidaway-Lee K, White E (2018). Continuity of care with doctors—a matter of life and death? A systematic review of continuity of care and mortality. BMJ Open.

[4] Baker R, Freeman GK, Haggerty JL (2020). Primary medical care continuity and patient mortality: a systematic review. Br J Gen Pract.

[5] Cabana MD, Jee SH (2004). Does continuity of care improve patient outcomes?. J Fam Pract.

[6] Worrall G, Knight J (2006). Continuity of care for older patients in family practice: how important is it?. Can Fam Physician.

[7] Rosano A, Loha CA, Falvo R (2013). The relationship between avoidable hospitalization and accessibility to primary care: a systematic review. Eur J Public Health.

[8] Jackson GL, Powers BJ, Chatterjee R (2013). Database of Abstracts of Reviews of Effects: Quality-assessed Reviews.

[9] Shi L, Starfield B, Politzer R, Regan J (2002). Primary care, self-rated health, and reductions in social disparities in health. Health Serv Res.

[10] Nicodemo C, McCormick B, Wittenberg R, Hobbs FR (2021). Are more GPs associated with a reduction in emergency hospital admissions? A quantitative study on GP referral in England. Br J Gen Pract.

[11] Fisher R, Dunn P, Asaria M, Thorlby R (2020). Level or not? Comparing general practice in areas of high and low socioeconomic deprivation in England. https://www.health.org.uk/sites/default/files/upload/publications/2020/LevelOrNot_Web1_0.pdf.

[12] Gershlick B, Fisher R (2019). A worrying cycle of pressure for GPs in deprived areas. https://www.health.org.uk/news-and-comment/blogs/a-worrying-cycle-of-pressure-for-gps-in-deprived-areas.

[13] Hart JT (1971). The inverse care law. Lancet.

[14] Barlow P, Mohan G, Nolan A, Lyons S (2021). Area-level deprivation and geographic factors influencing utilisation of general practitioner services. SSM Popul Health.

[15] Office for National Statistics (2019). How does UK healthcare spending compare with other countries?. https://www.ons.gov.uk/peoplepopulationandcommunity/healthandsocialcare/healthcaresystem/articles/howdoesukhealthcarespendingcomparewithothercountries/2019-08-29#how-much-does-the-uk-spend-on-healthcare-compared-with-its-international-peers.

[16] Radinmanesh M, Ebadifard Azar F, Aghaei Hashjin A (2021). A review of appropriate indicators for need-based financial resource allocation in health systems. BMC Health Serv Res.

[17] McLean G, Guthrie B, Mercer SW, Watt GCM (2015). General practice funding underpins the persistence of the inverse care law: cross-sectional study in Scotland. Br J Gen Pract.

[18] Fisher R (2021). 'Levelling up' general practice in England. https://www.health.org.uk/publications/long-reads/levelling-up-general-practice-in-england.

[19] Currie J, Boyce T, Evans L (2021). Life expectancy inequalities in Wales before COVID-19: an exploration of current contributions by age and cause of death and changes between 2002 and 2018. Public Health.

[20] Rhys G, Beerstecher HJ, Morgan CL (2010). Primary care capitation payments in the UK. An observational study. BMC Health Serv Res.

[21] StatsWales General practice population. https://statswales.gov.wales/Catalogue/Health-and-Social-Care/General-Medical-Services/General-practice-population.

[22] HM Treasury (2025). GDP Deflators at market prices, and money GDP. https://www.gov.uk/government/collections/gdp-deflators-at-market-prices-and-money-gdp.

[23] Wilkinson GN, Rogers CE (1973). Symbolic description of factorial models for analysis of variance. Appl Stat.

[24] NHS England (2024). Patients registered at a GP practice data quality statement. https://digital.nhs.uk/data-and-information/publications/statistical/patients-registered-at-a-gp-practice/data-quality-statement.

[25] Gibbons B, Thomas K (2023). The persistent inverse care law. https://www.bevanfoundation.org/views/the-persistant-inverse-care-law/.

[26] NHS England (2025). Appointments in general practice. https://digital.nhs.uk/data-and-information/publications/statistical/appointments-in-general-practice.

[27] NHS England Quality and Outcomes Framework. https://qof.digital.nhs.uk/.

[28] NHS England, Ipsos MORI (2024). GP Patient Survey. https://gp-patient.co.uk/surveysandreports.

[29] Beech J, Baird B (2020). GP funding and contracts explained. https://www.kingsfund.org.uk/insight-and-analysis/long-reads/gp-funding-and-contracts-explained.

